# Developing key performance indicators for the Canadian chiropractic profession: a modified Delphi study

**DOI:** 10.1186/s12998-022-00439-z

**Published:** 2022-08-01

**Authors:** Marc-André Blanchette, Silvano Mior, Shawn Thistle, Kent Stuber

**Affiliations:** 1 Department of Chiropratique, Université du Québec À Trois-Rivières (UQTR), Trois-Rivières, QC Canada; 2 Department of Research and Innovation, Canadian Memorial Chiropractic College (CMCC), Toronto, ON Canada

**Keywords:** Performance assessment, Profession: allied health, Quality assessment, Quality of care, Chiropractic, Delphi study

## Abstract

**Background:**

The purpose of this study is to develop a list of performance indicators to assess the status of the chiropractic profession in Canada.

**Method:**

We conducted a 4-round modified Delphi technique (March 2018–January 2020) to reach consensus among experts and stakeholders on key status indicators for the chiropractic profession using online questionnaires. During the first round, experts suggested indicators for preidentified themes. Through the following two rounds, the importance and feasibility of each indicator was rated on an 11-point Likert scale, and their related potential sources of data identified. In the final round, provincial stakeholders were recruited to rate the importance of the indicators within the 90th percentile and identified those most important to their organisation.

**Results:**

The first round generated 307 preliminary indicators of which 42 were selected for the remaining rounds, and eleven were preferentially selected by most of the provincial stakeholders. Experts agreed the feasibility of all indicators was high, and that data could be collected through a combination of data obtained from professional liability insurance records and survey(s) of the general population, patients, and chiropractors.

**Conclusions:**

A set of performance indicators to assess the status of the Canadian chiropractic profession emerged from a scientific and stakeholder consensus.

**Supplementary Information:**

The online version contains supplementary material available at 10.1186/s12998-022-00439-z.

## Background

Measuring performance is an important part of improvement in healthcare [1]. Reporting performance indicators helps to monitor function of a system, promote public accountability, and inform change management [1, 2]. Emerging evidence suggests that public reporting of performance indicators may stimulate providers to improve healthcare quality [3], although it is unclear whether these changes result in improved patient outcomes [4, 5].

In Canada, chiropractic is one of the most frequently sought nonphysician provider groups [6]. The Canadian annual utilisation of chiropractic services slightly increased from 10 to 11.7% between 1980 and 2015 [6]. The majority of chiropractors practice within private clinics [7], and since the delisting of chiropractic services in some provinces [8], administrative insurance billing data from most Canadian provincial health insurance plans are not available. Since most patients pay directly for their services [9], other public insurance plans like workers compensation boards and veteran affairs represent a marginal part of chiropractic activities [10]. Consequently, there are few quality or performance indicators that can be derived from Canadian public medico-administrative databases available to the chiropractic profession.

In response to this limitation in databases, the Canadian Chiropractic Association (CCA) began periodic surveying of their members in order to establish a “statistical portrait of chiropractors to be used in planning, evaluation and policy development” [9]. Unfortunately, the survey has been mainly used for administrative purposes and is not typically provided to the profession or the public. The survey is time-consuming (81 questions), and its response rate has steadily decreased from 70% in 1996 to 39% in 2011. Furthermore, the data is not easily accessible, and sparingly used in research projects [10].

Recent work suggests important knowledge-to-practice gaps in the care provided by chiropractors for musculoskeletal disorders [11, 12]. Public reporting of chiropractic care performance indicators could be an important component of clinical practice guideline (CPG) implementation [13] through a learning health system (LHS) [14] within the chiropractic profession. LHS is defined as a dynamic health ecosystem that synergistically aligns various dimensions of health care delivery and routinized cycles of continuing learning and improvement [14]. However, there is a need for consensus-based and credible indicators to evaluate the status of the chiropractic profession in Canada. A national, scientific, and widely endorsed list of indicators would be an important first step toward a rigorous and credible evaluation of the status of the Canadian chiropractic profession. The purpose of this study was to develop a list of indicators that could be used to assess the status of the chiropractic profession in Canada.

## Methods

We used a four round modified Delphi design [15] to identify the best indicators to assess the status of the chiropractic profession in Canada. In the first three rounds, we asked national chiropractic experts to rate each identified indicator by their importance, measurement feasibility, and best source of data acquisition. We conducted a fourth round involving key provincial and national association stakeholders to identify the most important indicators from their perspective. All responses were voluntary. Participants provided informed consent digitally before completing our questionnaires. Research ethics approval (#1802X03) was obtained from the Canadian Memorial Chiropractic College (CMCC).

### Participants

Participants were purposefully sampled from Canadian chiropractors. We defined “experts” as a Canadian chiropractor with a postgraduate degree in research (MSc and/or PhD). These criteria were used to ensure that the participants had sufficient knowledge of the Canadian chiropractic profession to identify relevant performance indicators. We invited stakeholders, representatives from each provincial and national chiropractic associations, provincial regulatory body and academic teaching institution. Eligible participants were identified by screening websites of relevant Canadian chiropractic organizations (provincial and national associations, foundations, regulatory bodies, and educational institutions). We also used snowball sampling [16]. Invitations to complete the survey were sent by email, with up to three weekly reminders for each round.

### Data collection

Data were collected for each of the first three rounds using online questionnaires distributed via SurveyMonkey® (www.surveymonkey.com, San Mateo, CA, USA) and administered by the Office of Research, CMCC. For the fourth round, feedback was obtained from a web-based survey tool developed by the Université du Québec à Trois-Rivières (UQTR) (https://confluence.uqtr.ca/display/AOPSP/BIQ).

### Round 1

Experts were provided ten open-ended questions and asked to identify potentially relevant indicators related to assessment of: (1) quality of care, (2) financial status, (3) use of chiropractic care, (4) inter-professional collaboration, (5) education of chiropractors, (6) research, (7) public perception, (8) legal status, (9) adverse events, and (10) chiropractor caseloads. Experts were also able to provide a list of relevant indicators that in their opinion, may not have been adequately captured by those listed in the survey. Two researchers (MAB and SM) independently analysed and converted responders’ answers from the first round into a preliminary list of 307 indicators that were subsequently grouped into nine main themes (Additional file 1). They then met with the full team to discuss and reach consensus on the approved themes to be distributed in Round 2.

### Round 2

Experts who completed the first round were invited to rank the importance of each preliminary list of identified indicators on an 11-point Likert rating scale from 0 (strongly disagree) to 10 (strongly agree). They were also asked to provide recommendations for any additional indicators not captured in Round 1, and suggest revisions to any of the proposed indicators. Indicators with median or mean importance scores in the 90th percentile were advanced to Round 3. To ensure that relevant aspects of the profession were measured, the indicators with the highest mean importance score for the themes that had less than two indicators in the 90th percentile were selected by the research team.

### Round 3

Experts who completed the first two rounds were invited to complete the third round. The mean and median importance scores obtained for each indicator at the end of Round 2 were included. Each expert evaluated the importance and feasibility of each indicator on an 11-point Likert rating scale and also identified the best source for the acquisition of data for each indicator from a list of ten suggested sources e.g., survey of: (1) chiropractic patients, (2) Canadian population, (3) chiropractors, (4) medical doctors; or data from: (5) provincial colleges of chiropractors, (6) Canadian Chiropractic Protective Association (CCPA) main Canadian chiropractic malpractice carrier, (7) private insurers, (8) provincial public health plan, (9) legal decision database, 10) CCA. The experts were also able to add non prespecified sources of data. All the indicators assessed during Round 3 were entered into the fourth round.

### Round 4

To consider the perspective of relevant stakeholders on the most important indicators, we invited the Executive Officers of the national and provincial chiropractic associations, regulatory boards, CCPA and Canadian Federation of Chiropractic, as well as representatives of chiropractic academic teaching institutions (CMCC and the UQTR). Each stakeholder was asked to rank the importance of each indicator from Round 3 on an 11-point Likert rating scale, and select the 15 most important indicators from the perspective of their organization/institution.

### Analysis

Descriptive statistics (mean, standard deviation, median, quartiles, mode) were used to assess the importance, feasibility and best source of data of the indicators. We used the independent samples Mann–Whitney U test to compare the importance score obtained during the third round and fourth round. All comparisons were considered statistically significant at p < 0.05 level. All analyses were performed using SPSS for Mac (version 26.0.0.1, IBM Corporation, Armonk, NY, USA).

## Results

We identified and invited 131 experts of whom 53 (40.5%) completed the first, 45 (84.9%) the second and 32 (71.1%) the third rounds. We invited 19 stakeholders to complete the fourth round, 10 (52.6%) completed the online survey but only 7 identified the most important indicators from the perspective of their organisation.

### Rounds 1 and 2

Round 1 produced 307 potential indicators that were grouped into 9 main themes and 32 subthemes (Additional file 1). The mean importance score for each indicator identified in Round 2 ranged between 3.1 and 9.2, with a mean of 7.5 (standard deviation (SD) of 0.9) (Additional file 1). Taking the 90th percentile of the mean and median importance score, we identified 34 indicators. Despite not being in the 90th percentile, the following 8 indicators were selected because they were rated as the two highest mean importance scores in their respective themes:

- Proportion of family health teams (academic or non-academic) including a chiropractor
- Proportion of medical doctors with positive attitude towards chiropractors/chiropractic services
- Number of hours spent on diagnosis
- Average cost per patient paid by Insurance coverage for chiropractic services
- Proportion of chiropractors that are satisfied with their job
- Proportion of chiropractors involved in multidisciplinary research
- Proportion of chiropractic researchers who conduct clinical research
- Legislated scope of practice in every province

The theme interprofessional collaboration had three indicators because *proportion of family health teams (academic or non-academic) including a chiropractor* and *proportion of medical doctors with positive attitude towards chiropractors/chiropractic services* had the same importance score. After Round 2, the indicator *proportion of family health teams (academic or non-academic) including a chiropractor* was reformulated prior to the third round to *proportion of family or community health teams (academic or non-academic) including a chiropractor.*

### Round 3

In the Round 3, the mean importance score ranged between 7.9 to 9.9, with a mean of 9.1 (SD = 0.5) (Table 1). The mean feasibility score ranged between 6.3 to 9.6 with a mean of 8.0 (SD = 0.8) (Table 1). The most common data sources reported to capture the indicators were: survey of chiropractors (31.0%), survey of chiropractic patients (26.2%), survey of the Canadian population (9.5%) and data from the CCPA (9.5%). After Round 3, the indicator *proportion of family or community health teams (academic or non-academic) including a chiropractor* was reformulated prior to the fourth round to *proportion of multidisciplinary medical clinics (e.g., family health teams, health teams *etc*.) academic or non-academic that include a chiropractor.*

**Table 1:** Expert’s evaluation of the indicators during the third round

Indicator	Importance					Feasibility					Source of data
	Median	Q1	Q3	Mean	SD	Median	Q1	Q3	Mean	SD	Mode
Measures related to utilization of care											
Proportion of chiropractic patients with neck pain‡	10.0	10.0	10.0	9.7	0.6	9.0	8.0	10.0	8.7	1.5	Patients survey
Proportion of chiropractic patients with back pain‡	10.0	10.0	10.0	9.8	0.6	9.0	8.0	10.0	8.8	1.4	Patients survey
Proportion of chiropractic patients with headache	10.0	9.0	10.0	9.4	0.9	9.0	8.0	10.0	8.6	1.4	Patients survey
Proportion of chiropractic patients with multiple musculoskeletal complaints	10.0	8.0	10.0	9.1	1.2	8.0	7.0	10.0	8.3	1.8	Patients survey
Proportion of chiropractic patients with acute conditions	9.0	8.0	10.0	8.7	1.6	8.0	7.0	10.0	8.1	1.8	Patients survey
Proportion of chiropractic patients with chronic conditions	10.0	8.0	10.0	9.0	1.3	8.0	7.0	10.0	8.4	1.5	Patients survey
Number of visits per episode of chiropractic care	9.0	8.0	10.0	8.9	1.3	8.0	7.0	9.0	8.0	1.8	Chiropractors survey
Proportion of third-party coverage per chiropractic service	9.0	8.0	9.0	8.4	1.3	9.0	7.0	10.0	8.3	1.8	Private Insurers
Proportion of the population that has extended health benefits covering chiropractic care	9.0	8.0	10.0	8.7	1.4	9.0	7.0	10.0	8.3	1.9	Private Insurers
Proportion of Canadians able to access chiropractic care	10.0	9.0	10.0	8.9	1.6	8.0	5.0	9.5	7.2	2.4	Population survey
Numbers of health facilities that include chiropractors in the health care team	9.0	8.0	10.0	8.2	2.3	8.0	6.8	9.0	7.8	1.8	Chiropractors survey
Measures related to inter-professional collaboration											
Proportion of patients referred by a medical doctor	9.0	7.0	9.0	8.2	1.8	8.0	7.0	10.0	7.7	2.1	Chiropractors survey
Proportion of family or community health teams (academic or non-academic) including a chiropractor†	9.0	8.0	10.0	8.5	1.4	8.0	7.0	9.0	7.8	1.7	Chiropractors survey
Proportion of medical doctors with positive attitude towards chiropractors/chiropractic services†	8.0	8.0	10.0	8.3	1.8	8.0	5.0	10.0	7.5	2.1	Physicians survey
Measures related to education											
Number of hours spent on diagnosis during chiropractic education†	9.0	8.0	10.0	8.8	1.4	10.0	9.8	10.0	9.6	0.7	Education institution
Number of hours spent on clinical training during chiropractic education	9.0	8.8	10.0	8.9	1.4	10.0	9.5	10.0	9.6	0.7	Education institution
Measures related to financial indicators											
Average cost per patient paid by Insurance coverage for chiropractic services†	8.0	8.0	10.0	8.5	1.3	9.0	8.0	10.0	8.7	1.5	Private Insurers
Proportion of chiropractors that are satisfied with their job†	8.0	7.0	10.0	8.1	1.6	9.0	8.0	10.0	8.6	1.6	Chiropractors survey
Measures related to outcomes and quality of patient care											
Proportion of chiropractors providing evidence-based care	10.0	9.0	10.0	9.5	0.7	7.0	6.0	8.0	6.9	1.9	Chiropractors survey
Proportion of chiropractors delivering patient-centred care	10.0	9.0	10.0	9.3	1.0	7.0	6.0	8.0	7.0	2.0	Chiropractors survey
Proportion of chiropractic patients who receive an appropriate physical exam	10.0	9.0	10.0	9.2	1.1	7.0	5.0	8.0	6.3	2.3	Chiropractors survey
Average proportion of chiropractic patients who receive first line recommendations (education. advice. self-care)	10.0	8.0	10.0	9.2	1.1	7.0	6.0	8.0	7.1	2.2	Chiropractors survey
Proportion of chiropractors who advise patients to stay active. return to ADL and work early	10.0	9.0	10.0	9.3	1.0	8.0	6.0	8.0	7.2	2.2	Chiropractors survey
Proportion of patients who receive advice to stay active. return to ADL and work early	10.0	9.0	10.0	9.3	1.0	8.0	5.0	9.0	7.2	2.3	Patients survey
Proportion of chiropractors who provide advice on exercise & physical activity	10.0	8.0	10.0	9.2	1.0	8.0	6.0	9.0	7.3	2.2	Chiropractors survey
Proportion of chiropractic patients who are satisfied with care	10.0	9.0	10.0	9.2	1.4	9.0	7.0	10.0	8.4	1.7	Patients survey
Proportion of chiropractic patients who experience a significant pain reduction	9.0	9.0	10.0	9.1	1.2	9.0	7.0	10.0	8.2	1.7	Patients survey
Proportion of chiropractic patients who experience a significant functional improvement	10.0	9.8	10.0	9.7	0.6	8.0	7.0	10.0	8.0	2.0	Patients survey
Proportion of injured workers who return to work within one month of chiropractic care	10.0	9.0	10.0	9.5	1.0	9.0	8.0	10.0	8.5	1.9	Worker compensation boards
Measures related to academic and research productivity											
Proportion of chiropractors involved in multidisciplinary research†	8.0	6.8	9.0	7.9	1.7	9.0	7.0	10.0	8.6	1.8	Chiropractors survey
Proportion of chiropractic researchers who conduct clinical research†	8.5	7.0	9.3	8.2	1.6	10.0	8.0	10.0	8.8	1.8	Chiropractors survey
Measures related to marketing. professionalism and public perception											
Proportion of chiropractors who use X-rays for assessment and reassessment	9.0	8.0	10.0	8.5	1.9	8.0	7.0	10.0	7.7	2.2	Chiropractors survey
Proportion of chiropractors that use unethical billing procedures	10.0	9.0	10.0	9.1	1.8	6.0	5.0	9.0	6.4	2.6	College of chiropractors
Proportion of the population that perceives chiropractic care as valuable type of care	10.0	9.0	10.0	9.1	1.6	8.0	7.0	10.0	8.1	1.9	Population survey
Proportion of the population that perceives chiropractors as credible healthcare	10.0	9.0	10.0	9.3	1.6	8.0	7.0	10.0	8.1	2.0	Population survey
Proportion of the population who trust the chiropractic profession‡	9.5	8.0	10.0	9.0	1.6	8.0	7.0	10.0	8.1	2.0	Population survey
Measures related to regulation and legal matters											
Legislated scope of practice in every province†	10.0	8.0	10.0	9.0	1.5	10.0	9.0	10.0	9.6	0.8	College of chiropractors
Number of provincial jurisdictions where chiropractors are recognized as healthcare professional by the healthcare system	10.0	9.0	10.0	8.9	1.7	10.0	9.0	10.0	9.2	1.4	College of chiropractors
Adverse events											
Proportion of chiropractic patients who experienced severe adverse events b	10.0	10.0	10.0	9.8	0.6	8.0	6.0	10.0	7.7	2.1	CCPA
Proportion of adverse events needing urgent medical attention (e.g. hospitalization) ‡	10.0	10.0	10.0	9.7	0.7	8.0	6.0	10.0	7.5	2.3	CCPA
Proportion of adverse events resulting in permanent impairment‡	10.0	10.0	10.0	9.6	0.8	8.0	6.0	10.0	7.6	2.3	CCPA
Proportion of adverse events resulting in patient death‡	10.0	10.0	10.0	9.8	0.6	8.0	6.0	10.0	7.8	2.3	CCPA

^†^ > 90th percentile during round 2

^‡^Importance score significantly higher than in round 2 (p < 0.05)

CCPA: Canadian chiropractic protective association

SD: Standard deviation

Q1: First quartile

Q3: Third quartile

### Round 4

During the fourth round, the mean importance score ranged between 6.5 to 9.3 with a mean of 8.2 (SD = 0.8) (Table 2). Most of the importance scores were not significantly different from Round 3, except for nine indicators that were ranked significantly lower. Seven of the ten stakeholders that responded in Round 4 ranked their 15 most important indicators. The following eleven indicators were selected by more than 50% of the stakeholders:

- Proportion of chiropractic patients with neck pain
- Proportion of chiropractic patients with back pain
- Proportion of chiropractic patients with headache
- Proportion of chiropractic patients with chronic conditions
- Proportion of chiropractors providing evidence-based care
- Proportion of chiropractors delivering patient-centred care
- Proportion of chiropractic patients who receive an appropriate physical exam
- Proportion of chiropractic patients who experience a significant functional improvement
- Proportion of the population that perceives chiropractors as credible healthcare providers
- Proportion of the population who trust the chiropractic profession
- Proportion of chiropractic patients who experienced severe adverse events

**Table 2:** Stakeholder’s evaluation of the indicators during the fourth round

Indicator	Importance					Comparison with round 3 (p-value)	Selection of most important indicators (%)
	Median	Q1	Q3	Mean	SD		
Measures related to utilization of care							
Proportion of chiropractic patients with neck pain†	8.5	8.0	10.0	8.8	0.9	0.009	**57**
Proportion of chiropractic patients with back pain†	9.0	8.0	10.0	9.1	0.9	0.030	**57**
Proportion of chiropractic patients with headache	8.5	7.8	10.0	8.5	1.4	0.070	**57**
Proportion of chiropractic patients with musculoskeletal complaints	9.5	8.8	10.0	9.3	0.8	0.929	43
Proportion of chiropractic patients with acute conditions	8.0	6.8	9.3	8.0	1.5	0.156	43
Proportion of chiropractic patients with chronic conditions	8.5	7.8	10.0	8.5	1.4	0.274	**57**
Number of visits per episode of chiropractic care†	6.0	5.0	9.0	6.9	2.1	0.010	29
Proportion of third-party coverage per chiropractic service	7.5	5.8	9.0	7.0	2.4	0.070	43
Proportion of the population that has extended health benefits covering chiropractic care†	7.0	4.8	8.3	6.5	2.4	0.002	29
Proportion of Canadians able to access chiropractic care†	8.5	5.0	9.3	7.5	2.3	0.043	43
Number of health facilities (hospitals. senior care. hospice. etc.) that include chiropractors in the health care team	9.0	2.5	10.0	6.5	4.0	0.481	14
Measures related to inter-professional collaboration							0
Proportion of patients referred by a medical doctor	7.5	5.3	9.0	6.9	2.3	0.062	14
Proportion of multidisciplinary medical clinics (e.g. family health teams. health teams. etc.). academic or non-academic that include a chiropractor	8.0	5.8	9.0	7.6	1.8	0.123	43
Proportion of medical doctors with positive attitude towards chiropractors/chiropractic services	7.5	5.8	10.0	7.6	2.2	0.362	43
Measures related to education							0
Number of hours spent on diagnosis during chiropractic education	9.0	7.0	10.0	8.4	1.6	0.555	29
Number of hours spent on clinical training during chiropractic education	9.0	6.5	10.0	8.0	2.2	0.240	43
Measures related to financial indicators							0
Average cost per patient paid by Insurance coverage for chiropractic services	8.0	5.0	9.0	7.3	2.1	0.207	0
Proportion of chiropractors that are satisfied with their job	7.0	5.5	8.5	7.1	1.5	0.109	0
Measures related to outcomes and quality of patient care							0
Proportion of chiropractors providing evidence-based care†	8.0	7.0	9.5	8.1	1.5	0.006	**71**
Proportion of chiropractors delivering patient-centred care	9.0	8.0	10.0	8.9	1.1	0.298	**71**
Proportion of chiropractic patients who receive an appropriate physical exam	10.0	8.0	10.0	9.1	1.2	0.786	**71**
Average proportion of chiropractic patients who receive first line recommendations (education. advice. self-care)	9.0	5.5	10.0	8.1	2.2	0.250	14
Proportion of chiropractors who advise patients to stay active. return to ADL and work early to their patients†	9.0	6.5	9.5	8.1	1.8	0.042	0
Proportion of patients who receive advice to stay active. return to ADL and work early†	9.0	6.5	9.5	8.1	1.8	0.034	14
Proportion of chiropractors who provide advice on exercise & physical activity†	9.0	6.0	9.5	8.0	1.7	0.042	0
Proportion of chiropractic patients who are satisfied with care	10.0	8.0	10.0	9.1	1.2	0.703	43
Proportion of chiropractic patients who experience a significant pain reduction	10.0	8.0	10.0	9.1	1.2	0.892	43
Proportion of chiropractic patients who experience a significant functional improvement	10.0	9.0	10.0	9.3	1.0	0.378	**57**
Proportion of injured workers who return to work within one month of chiropractic care	9.0	8.0	10.0	8.8	1.3	0.097	29
Measures related to academic and research productivity							0
Proportion of chiropractors involved in multidisciplinary research	8.0	5.0	9.0	7.1	2.1	0.334	14
Proportion of chiropractic researchers who conduct clinical research	8.0	6.0	9.0	7.6	1.5	0.280	0
Measures related to marketing. professionalism and public perception							0
Proportion of chiropractors who overuse X-rays for assessment and re-assessment	7.0	6.0	10.0	7.6	2.1	0.271	29
Proportion of chiropractors that use unethical billing procedures	8.0	7.5	10.0	8.2	2.3	0.238	14
Proportion of the population that perceives chiropractic care as valuable type of care	10.0	6.5	10.0	8.6	1.8	0.495	29
Proportion of the population that perceives chiropractors as credible healthcare providers	10.0	8.0	10.0	8.9	1.5	0.478	**57**
Proportion of the population who trust the chiropractic profession	9.0	8.0	10.0	8.8	1.4	0.530	**57**
Measures related to regulation and legal matters							0
Legislated scope of practice in every province	10.0	9.0	10.0	9.1	2.0	0.476	43
Number of provincial jurisdictions where chiropractors are recognized as healthcare professional by the healthcare system	10.0	6.0	10.0	8.2	2.2	0.549	29
Adverse events							0
Proportion of chiropractic patients who experienced severe adverse events	10.0	9.0	10.0	8.9	2.4	0.357	**71**
Proportion of adverse events needing urgent medical attention (e.g. hospitalization)	9.5	8.0	10.0	8.5	2.4	0.121	29
Proportion of adverse events resulting in permanent impairment	9.5	8.0	10.0	8.3	3.1	0.190	29
Proportion of adverse events resulting in patient death	10.0	9.0	10.0	8.6	3.1	0.223	29

^†^Importance score significantly lower than in round 3 (p < 0.05)

Bold: Indicator selected by more than 50% of stakeholders

SD: Standard deviation

Q1: First quartile

Q3: Third quartile

## Discussion

Our findings support a recently described theoretically based integrated framework for healthcare performance assessment [17]. The indicators most frequently selected by our stakeholders reflect the framework’s measurable constructs of patients’ needs (proportion of chiropractic patients with headache, chronic conditions, neck and back pain) and expectations (proportion of the population that trust the chiropractic profession and that perceives chiropractors as credible healthcare providers); receipt and experience of healthcare (proportion of chiropractic patients who receive an appropriate physical exam, proportion of chiropractors providing evidence-based care, and patient-centred care); and healthcare outcomes (proportion of chiropractic patients who experience a significant functional improvement, and severe adverse events). Consistent with the framework, the combination of these indicators could provide insight on accessibility, appropriateness, effectiveness, and safety of chiropractic care [17]. Our experts considered these indicators to be of high importance and feasible to collect using a combination of professional liability insurance records and surveys of the general population, patients, and chiropractors. The responding stakeholders confirmed the high importance of most of these indicators. They also reflect the contemporary emphasis on quality of care [11, 12], patient-centred care [18], safety [19], and public legitimacy[20] within the chiropractic profession.

To our knowledge this is the first study to identify key performance indicators to assess the status of the Canadian chiropractic profession. Others have identified multiple quality indicators for the management of musculoskeletal disorders in emergency departments [21]. However, these indicators may not be directly applicable as they involve care pathways and pharmacological treatment not commonly used in chiropractic care. Sorensen et al*.* have developed disease-specific quality indicators for Danish chiropractic patients with low back pain [13]. Their focus was narrower than ours, but their work suggests that it is feasible to measure performance using indicators obtained by surveying chiropractors. More recently, Dutch physiotherapists have measured a core set of healthcare outcomes from routinely collected clinical patient data (patient reported outcomes measures [PROMs]) [22, 23]. Wide implementation of this core set appears promising since the stakeholders involved in collecting these outcomes realized they added value to their clinical practice [22].

Since previous studies have demonstrated that it is feasible to derive performance indicators from both patient and practitioner surveys [13, 22], we argue in favor of regular public reporting of performance indicators for the chiropractic profession in Canada. Developing an adequate infrastructure for data collection will be challenging given the multiple sources of information required. Considering that provincial and national chiropractic organizations (associations and regulatory boards) regularly survey their members and the population, this data provides an opportunity to optimize and harmonize resources, and contribute to performance metrics. However, collecting survey data to inform performance indicators will require optimizing survey methods to ensure a sufficient response rate to mitigate potential bias [24]. Both the CCA and the Canadian Chiropractic Federation, by nature of their national positions, can be strategic players in rallying provincial organizational participation. Chiropractic regulatory boards periodically audit their members regarding their quality of care within the context of their mandate of public protection. This might be an appropriate context to collect data on the receipt and experience of healthcare. Electronic health records might also facilitate the collection of chiropractors’ and patients’ data [25–27].

Negative unintended consequences have been reported following the public reporting of performance indicators [28, 29]; however, the potential for quality improvement is considered to outweigh the risk [1, 3]. Public reporting of performance indicators is uncommon among chiropractic organizations and could potentially increase their transparency and accountability while facilitating the implementation of a LHS.

Among the strengths of our study is the participation from experts and national stakeholders across Canada. Most of the suggested indicators were identified as important by our experts, which may lead to an overabundance of information (“indicator chaos”) [30]. Our study has limitations, such that the stakeholders’ perception of importance of indicators omitted two constructs (healthcare resources and structures, and healthcare processes, functions and context) that would have been highlighted through a theory-based selection [17]. Moreover, the indicators identified in relation to the quality of care require further development to be adequately operationalized [13, 31]. Although our response rate is common among healthcare provider surveys, it is not optimal and suggests that mobilizing stakeholders toward the measurement and public reporting of indicators may be challenging.

## Conclusion

We present a set of performance indicators for the Canadian chiropractic profession developed from a consensus of scientific experts and stakeholders. This set of indicators constitute a promising basis for the assessment of the chiropractic profession’s status. Further development regarding the measurement quality of the indicators, the supporting infrastructure, and the stakeholder’s engagement will be necessary prior to implementation.

## Supplementary Information

**Additional file 1:** Expert’s evaluation of the indicators during the first and second rounds.

## Data Availability

The datasets generated and analyzed during the current study are not publicly available due privacy restriction but are available from the corresponding author on reasonable request.

## Abbreviations

CCA: Canadian Chiropractic Association
CCPA: Canadian Chiropractic Protective Association
CMCC: Canadian Memorial Chiropractic College
CPG: Clinical Practice Guidelines
LHS: Learning Health System
PROMs: Patient reported outcome measures
Q1: First quartile
Q3: Third quartile
SD: Standard Deviation
UQTR: Université du Québec à Trois-Rivières
